# Predictive factors for sentinel node metastases in primary invasive breast cancer: a population-based cohort study of 2552 consecutive patients

**DOI:** 10.1186/s12957-018-1353-2

**Published:** 2018-03-12

**Authors:** Shabaz Majid, Lisa Rydén, Jonas Manjer

**Affiliations:** 1 Department of Surgery, Central Hospital of Kristianstad, SE-291 85 Kristianstad, Sweden; 2 Department of Clinical Sciences Malmö, Lund University, Malmö, Sweden; 3 Department of Surgery, Skåne University Hospital, Malmö, Sweden; 4 Department of Clinical Sciences Lund, Lund University, Lund, Sweden

**Keywords:** Invasive breast cancer, Predictive factors, Sentinel node metastases

## Abstract

**Background:**

Axillary lymph node status is one of the most important prognostic factors for breast cancer. The aim of this study was to determine predictive factors for metastasis to sentinel node (SN) in primary invasive breast cancer.

**Method:**

This is a study of 3979 patients with primary breast cancer during 2008–2013 in Malmö and Lund scheduled for surgery and included in the information retrieved from Information Network for Cancer Care (INCA). The final study population included 2552 patients with primary invasive breast cancer. The risk of metastases to SN were examined in relation to potential clinicopathological factors such as age, screening mammography, tumor size, tumor type, histological grade, estrogen status, progesterone status, Her-2 status, multifocality, and lymphovascular invasion. Binary logistic regression was used; adjusted analyses yielded odds ratio (OR) with 95% confidence interval.

**Results:**

Tumors detected by mammography screening were less likely to be associated with metastases to SN compared to those not found by mammography screening (0.63; 0.51–0.80). Negative hormonal status for estrogen associated with lower risk for SN metastases compared to tumor with positive estrogen status (0.64; 0.42–0.99). Tumors with a size more than 20 mm had higher risk to metastasize to SN (1.84; 1.47–2.33) compared to tumors less than 20 mm. Multifocality (1.90; 1.45–2.47) and lymphovascular invasion (3.74; 2.66–5.27) were also strong predictive factors for SN metastases.

**Conclusion:**

SN metastasis is less likely to occur in women with invasive breast cancer diagnosed by screening mammogram. Tumors with negative estrogen status are associated with low risk for SN metastases. Tumors larger than 20 mm, multifocality, or lymphovascular invasion are also factors associated with high risk for SN metastases.

## Background

Axillary lymph node status is still one of the most important prognostic factors for predicting clinical outcome in invasive breast cancer [1, 2], and it also determines the extent of axillary surgery and adjuvant/systemic therapy. Recently, the value of an axillary clearance when metastatic spread is found has been questioned [3, 4]. Indeed, it may be questioned if staging is necessary in all cases, e.g., even in patients where the risk of metastatic spread is very low. However, this demands that low-risk groups can be accurately identified [5].

Physical examination is a poor predictor of axillary lymph node metastasis [6], and evaluation of the axilla by ultrasound has been shown to be unreliable [7].

Sentinel node biopsy (SNB) has been used since the late 1990s to evaluate the axillary lymph node status [1]. The sentinel lymph node is defined as the first lymph nodes to which cancer cells are most likely to spread from the primary tumor. SNB has minimized the need for axillary lymph node dissection (ALND) dramatically which in turn decreases the subsequent complications after ALND such as lymphedema, chronic pain, and neurological disabilities [8, 9]. Many studies have confirmed that SNB is technically feasible, safe, and associated with fewer complications as compared with ALND [1, 2, 9]. However, the SNB as a process is time consuming and resource intensive and, in the majority of patients, SNs are without metastases, and although the SNB is associated with fewer complications, there is still a risk to develop disabilities postoperatively [9, 10]. The proportion of T1 invasive breast carcinomas is increasing due to factors such as better diagnostic methods and public screening programs, and the role of SNB and ALND in these patients has been questioned [3, 11, 12].

The aim of this study was to define clinical and pathological factors that predict patients who are likely to be node-positive and thus to have the possibility for better planning of surgical or systemic therapy. Moreover, the information can enable the identification of patients with a high probability of node-negative tumor where the SN procedure may possibly be omitted.

## Methods

The background population consists of all cases of breast cancer among women in Lund and Malmö operated on between January 2008 and December 2013. Every patient was identified by a 12-digit civil registration number which is unique for every Swedish citizen. All patients operated on because of breast cancer were included, and a total number of 3979 cases (cancer events) were identified. The indication for SNB has been changed over the time; in the early beginning of 2000s, the SNB procedure was performed only when tumor size was smaller than 30 mm and all cases with tumor size larger than 30 mm as well as multifocal tumors underwent ALND directly in both centers.

The following patients were excluded: 30 male patients, 82 cases with bilateral breast cancer (that is, 164 cancer events), 43 cases with previous breast cancer, and 1040 cases who were not operated on with SNB; 122 cases were diagnosed with in situ breast cancer and 25 patients who had received systemic therapy preoperatively. In two patients, it was not known if they had received systemic therapy, and one patient had unknown information about SN status. Regarding those cases not operated on with SNB (1040 patients), there were 599 who underwent an axillary dissection; 191 women had neither underwent SNB nor an ALND, and information on axillary surgery was missing in the remaining 250 patients.

In the 1040 excluded patients, 48.5% had tumors that were stage T2 or above; among ALND operated cases, this percentage was 35.3%; and in women with neither SNB nor ALND was 1.25%. The corresponding percentage in our study population of 2552 patients was 22.8%.

**Fig. 1:**
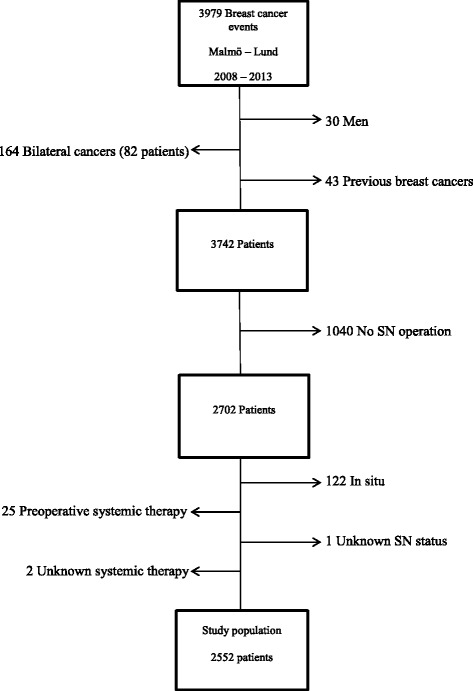
Patient selection

Furthermore, among all 1040 excluded patients, 18.7% had a multifocal tumor. In ALND cases, this proportion was 16.7%; in patients with no surgery in the axilla, it was 1.8%; and in our study population, it was 13.8%. Following these exclusions, the final study population included 2552 patients (Fig. 1).

In Sweden, a nationwide database for breast cancer is available on an IT platform called The Information Network for Cancer Care (INCA). INCA manages various information about cancer care as well as long-term follow-up. It is run and developed jointly by the country’s regional cancer centers. INCA has been in full operation since 2007. The Regional Cancer Center in Southern Sweden (RCC-Syd) is the main center in the southern area which manages this registry.

In Malmö and Lund at Skåne University Hospital, every patient with breast cancer is reviewed and discussed before and after surgery at a weekly multidisciplinary breast cancer conference at which there are representatives from the departments of oncology, radiology, surgery, and pathology. A special registration form designed by INCA is available, and this form is filled in by a surgeon in cooperation with a secretary who is specifically employed for this reason and who is responsible for entering the data into the platform. The present study was approved by the Ethic Committee at Lund University, Lund, Sweden (LU-Dnr 2013/821).

All information used in the present study such as screening, age, menopause status, tumor size, histopathological type and grade, receptor status, Her-2, multifocality, and lymphovascular invasion as well as information about SNB, i.e., type and size of metastases, were retrieved from INCA.

All women in the background population aged 40–74 years are invited to the public mammography screening. Mode of detection was recorded as screening-detected yes/no. Menopause status was defined as premenopausal or postmenopausal.

Postmenopausal women were subdivided as to when they had their last menstruation, 6 months to 5 years ago or more than 5 years after menopause. Tumor size was defined according to the TNM classification, T1 tumor ≤ 20 mm, T2 tumor 21–50 mm, and T3–T4 > 50 mm. [13]. The T1 tumors were further classified to sub-groups T1a 1–5 mm, T1b 6–10 mm, and T1c 11–20 mm. The histopathological types were classified according to the WHO classification system; this system describes mainly six different histopathological types of invasive cancer [13–15]. We merged this into four different groups, i.e., ductal, lobular, combined ductal with lobular, and other rare types. Histological grade was defined according to the Nottingham histological grading (NHG) [16]. Multifocal tumors were defined as two or more tumors with normal tissue or in situ tumors at a distance of at least 20 mm. Lymphovascular invasion is defined as tumor cells in vascular spaces, tumor cells in underlying endothelium of vascular channels, and tumor cells invading through a vessel wall and endothelium [17]. Receptor status for both estrogen and progesterone was measured by immunohistochemistry (IHC). Receptor percentage more than 10% was regarded as positive and those with 10% or less as negative [18]. Her-2 protein was analyzed with IHC, and test results were reported as 0, 1+, 2+, or 3+. Fluorescent in situ hybridization (FISH) uses fluorescent pieces of DNA that specifically stick to copies of the HER2 gene in cells. In all cases, IHC test was used first and then completed by FISH in certain cases where Her-2 was 2+ or 3+. Her-2 status was classified as negative when Her-2 IHC = 0–1+ and 2+ in non-amplified tumors. Her-2 was regarded as positive if Her-2 was classified as 2+ or 3+ and amplified by FISH [19] 

Metastases in SN were classified as macrometastases when the size was > 2 mm and regarded as micrometastases when the size was 0.2–2.0 mm. All metastases with a size less than 0.2 mm were regarded as sub-micrometastases. Lymph nodes with only sub-micrometastases also referred to as isolated tumor cells (ITCs) were regarded as without metastases in the present analysis [18].

Lymph nodes with macro- or micrometastases were regarded as positive and those without metastases as negative. To compare the association between potential predictive factors and metastases in SN, binary logistic regression was used and all analyses were adjusted for all included factors, i.e., screening, age, tumor size, menstrual status, histopathological type and grade, receptor status for estrogen and progesterone, Her-2 status, presence of multifocality, and presence of vascular invasion. This yielded odds ratios (ORs) with 95% confidence intervals (CIs). Main analyses were performed for the two centers together, center A (Lund) and center B (Malmö). Moreover, the analyses were also performed for each center separately. Statistical Package for the Social Sciences (SPSS) program version 22.0 (SPSS Institute, Chicago, IL, USA) was used for all analyses.

## Results

There were in total 671 patients with SN metastases (26.3%), three hundred seventy four patients (29.6%) in center A and 297 patients (23.0%) in center B. Tumors detected by mammography screening were less likely to be associated with SN metastases compared with those not found by screening mammography (0.63; 0.51–0.80), Tables 1 and 2. A tumor with a size more than 20 mm, i.e., T2, T3, and T4, had higher possibility to metastasize to the SN compared to tumors less than 20 mm (T1): 1.84 and 1.47–2.33 for T2 and 2.56 and 1.07–6.09 for T3 and T4. An additional analysis showed that T1a tumors had the lowest risk for SN metastases (0.19; 0.09–0.40) followed by T1b (0.46; 0.34–0.63) compared with T1c.

**Table 1:** Potential predictive factors in relation to SN status

Determinants	Category	Total	SN negative		SN positive	
			*N*	%	*N*	%
Screening	No	1062	719	38.2	343	51.1
	Yes	1435	1123	59.7	312	46.5
	Unknown	55	39	2.1	16	2.4
Age	≤ 50	499	337	17.9	162	24.1
	51–74	1702	1290	68.6	412	61.4
	≥75	351	254	13.5	97	14.5
Menopause status	Pre	512	348	18.5	164	24.4
	Post < 5 years	228	168	8.9	60	8.9
	Post ≥ 5 years	1721	1299	69.1	422	62.9
	Unknown	91	66	3.5	25	3.7
Tumor size	T1	1505	1138	60.5	367	54.7
	T2	559	346	18.4	213	31.7
	T3 and T4	25	13	0.7	12	1.8
	Unknown	463	384	20.4	79	11.8
Tumor type	Ductal	1866	1324	70.4	542	80.8
	D and L	52	35	1.9	17	2.5
	Lobular	304	216	11.5	88	13.1
	Other	330	306	16.3	24	3.6
Histological grade	I	622	488	25.9	134	20.0
	II	1112	807	42.9	305	45.5
	III	790	563	29.9	227	33.8
	Unknown	28	23	1.2	5	0.7
Estrogen receptor	Positive	2144	1538	81.8	606	90.3
	Negative	279	218	11.6	61	9.1
	Unknown	129	125	6.6	4	0.6
Progesterone receptor	Positive	1851	1320	70.2	531	79.1
	Negative	571	436	70.2	531	79.1
	Unknown	130	125	6.6	5	0.7
HER-2 status	Negative	1466	1041	55.3	423	63.0
	Positive	240	174	9.3	66	9.8
	Unknown	848	666	35.4	182	27.1
Multifocality	No	1570	1184	62.9	386	57.5
	Yes	352	208	11.1	144	21.5
	Unknown	630	489	26.0	141	21.0
Vascular invasion	No	1324	1056	56.1	268	39.9
	Yes	184	87	4.6	97	14.5
	Unknown	1044	738	39.2	306	45.6

**Table 2:** Potential predictive factors and risk of SN metastases

Determinants	Category	SN negative	SN positive	OR 95% CI	OR 95% CI^a^
Screening	No	719	343	1.00	1.00
	Yes	1123	312	0.59(0.49–0.70)	0.63(0.51–0.80)
	Unknown	39	16	0.86(0.48–1.57)	0.88(0.46–1.66)
Age	≤ 50	337	162	1.00	1.00
	51–74	1290	412	0.67(0.53–0.82)	0.92(0.63–1.37)
	≥ 75	254	97	0.80(0.59–1.08)	0.70(0.42–1.11)
Menopause Status	Pre	348	164	1.00	1.00
	Post < 5 years	168	60	0.76(0.53–1.08)	0.98(0.62–1.53)
	Post ≥ 5years	1299	422	0.69(0.56–0.86)	0.82(0.56–1.22)
	Unknown	66	25	0.80(0.49–1.32)	0.89(0.50–1.53)
Tumor size	T1	1138	367	1.00	1.00
	T2	346	213	1.91(1.56–2.34)	1.84(1.47–2.33)
	T3 and T4	13	12	2.87(1.30–6.32)	2.56(1.07–6.09)
	Unknown	384	79	0.63(0.49–0.83)	0.67(0.50–0.93)
Tumor type	Ductal	1325	542	1.00	1.00
	D and L	35	17	1.19(0.66–2.13)	1.01 (0.54–1.90)
	Lobular	217	88	1.00(0.77–1.30)	0.87(0.64–1.20)
	Others	306	24	0.20(0.12–0.30)	0.29(0.18–0.46)
Histological grade	I	488	134	1.00	1.00
	II	807	305	1.37(1.09–1.73)	1.02(0.80–1.31)
	III	563	227	1.46(1.14–1.87)	1.10(0.82–1.50)
	Unknown	23	5	0.79(0.29–2.12)	1.40(0.46–4.31)
Estrogen receptor	Positive	1538	606	1.00	1.00
	Negative	218	61	0.71(0.52–0.96)	0.64(0.42–0.99)
	Unknown	125	4	0.09(0.03–0.22)	0.06(0.00–0.82)
Progesterone receptor	Positive	1320	531	1.00	1.00
	Negative	436	135	0.77(0.61–0.96)	0.78(0.56–1.07)
	Unknown	125	5	0.10(0.04–0.24)	3.80(0.30–47.42)
Her-2 status	Negative	1041	423	1.00	1.00
	Positive	174	66	0.93(0.69–1.27)	0.84(0.60–1.20)
	Unknown	666	182	0.68(0.56–0.82)	0.98(0.78–1.24)
Multifocality	No	1184	386	1.00	1.00
	Yes	208	144	2.12(1.67–2.70)	1.90(1.45–2.47)
	Unknown	489	141	0.89(0.71–1.10)	0.86(0.67–1.09)
Vascular invasion	No	1056	268	1.00	1.00
	Yes	87	97	4.40(3.20–6.04)	3.74(2.66–5.27)
	Unknown	738	306	1.63(1.36–1.98)	2.10(1.68–2.62)

^a^Adjusted including screening, age, menopause status, tumor size, tumor type, histological grade, estrogen status, progesterone status, Her-2 status, multifocality, and vascular invasion

**Table 3:** Potential predictive factors and risk of SN metastases separately for center A and center B

Determinants	Category	Center A			Center B		
		SN positive	SN negative	OR 95% CI	SN positive	SN negative	OR 95% CI
Screening	No	297	179	1.00	422	164	1.00
	Yes	552	179	0.62(0.46–0.86)	571	133	0.63(0.47–0.88)
	Unknown	39	16	0.82(0.42–1.60)	–	–	–
Age	≤ 50	157	86	1.00	180	76	1.00
	51–74	620	237	1.02(0.59–1.78)	670	175	0.82(0.48–1.44)
	≥ 75	111	51	0.69(0.34–1.39)	143	46	0.69(0.34–1.37)
Menopause status	Pre	167	90	1.00	181	74	1.00
	Post < 5 years	91	29	0.67(0.34–1.29)	77	31	1.32(0.70–2.50)
	Post ≥ 5 years	589	238	0.88(0.50–1.52)	710	184	0.77(0.43–1.34)
	Unknown	41	17	0.81(0.40–1.67)	25	8	0.87(0.34–2.16)
Tumor size	T1	394	191	1.00	744	176	1.00
	T2	136	105	1.63(1.16–2.30)	210	108	2.13(1.54–2.94)
	T3 and T4	4	7	6.28(1.50–26.40)	9	5	1.48(0.44–4.90)
	Unknown	354	71	0.60(0.42–0.84)	30	8	1.70(0.72–4.04)
Tumor type	Ductal	620	306	1.00	704	236	1.00
	D and L	25	12	0.89(0.42–1.88)	10	5	1.20(0.38–3.79)
	Lobular	80	45	0.90(0.58–1.40)	136	43	0.84(0.54–1.30)
	Others	163	11	0.23(0.11–0.50)	143	13	0.30(0.17–0.57)
Histological grade	I	218	66	1.00	270	68	1.00
	II	363	169	1.31(0.91–1.89)	444	136	0.80(0.56–1.16)
	III	290	136	1.49(0.98–2.26)	273	91	0.80(0.51–1.24)
	Unknown	17	3	1.30(0.31–5.37)	6	2	2.38(0.36–15.87)
Estrogen receptor	Positive	679	335	1.00	859	271	1.00
	Negative	98	35	0.59(0.33–1.04)	120	26	0.72(0.38–1.40)
	Unknown	111	4	0.07(0.00–1.04)	14	–	–
Progesterone receptor	Positive	578	290	1.00	742	241	1.00
	Negative	199	79	0.71(0.48–1.08)	237	56	0.79(0.50–1.23)
	Unknown	111	5	4.15(0.31–54.96)	14	–	–
HER-2	Negative	630	292	1.00	411	131	1.00
	Positive	77	44	1.04(0.66–1.64)	97	22	0.63(0.37–1.10)
	Unknown	181	38	0.93(0.59–1.50)	485	144	1.04(0.77–1.42)
Multifocality	No	452	194	1.00	732	192	1.00
	Yes	93	71	1.58(1.07–2.32)	115	73	2.21(1.50–3.23)
	Unknown	343	109	0.72(0.53–0.99)	146	32	0.88(0.56–1.40)
Vascular invasion	No	148	41	1.00	908	227	1.00
	Yes	18	34	6.10(2.98–12.50)	69	63	3.04(2.03–4.57)
	Unknown	722	299	1.64(1.10–2.44)	16	7	1.80(0.66–4.93)

A negative association with SN metastases was seen in cases with negative hormonal status for estrogen (0.64; 0.42–0.99). Multifocal tumors (1.90; 1.45–2.47) or tumors with vascular invasion (3.74; 2.66–5.27) had higher risk of SN metastases. Tumor types other than ductal and lobular, i.e., medullary and other rare types, were associated with low risk for SN metastases (0.29; 0.18–0.46). Adjusted analyses were similar to crude values except for histological grade II and III where crude analyses were associated with higher risk for SN metastases, but this was not observed with adjusted analyses. Overall, there were no large differences in results between the two centers; however, the risk of metastases to SN in all T3 and T4 cases were not high in center B while in center A there was a high risk of SN metastases in T3 and T4 cases (Table 3). In all analyses, there was no statistical significance in different histological grades in both centers, and hormonal status was not statistically significant when we analyzed each center separately. These analyses included few cases and CI was relatively wide.

## Discussion

In this study, we identified predictive factors for SN metastases by analyzing clinical and pathological characteristics of the tumors in patients with primary invasive breast cancer, and we found that SN metastasis is less likely to occur in women diagnosed by screening mammography. Tumors with negative estrogen status were associated with low risk of SN metastases. Tumors with a size more than 20 mm, multifocality, or lymphovascular invasion had more risk for SN metastases.

The strengths of the present study include the size of the sample, where 3979 patients with breast cancer were included from a non-selected population-based cohort of consecutive cases. Those patients who did not undergo SNB procedure and were excluded in final study population, they were mainly divided into two groups, carcinoma in situ and advanced invasive tumors where majority were T2 tumors. The reliability of collected data and accuracy of registration might be questioned; however, the quality of the INCA registry is regarded as very high with periodic validation control of data recording [20].

Assessment of axillary lymph status is essential because it predicts the clinical outcome and it also determines the extent of axillary surgery and adjuvant/systemic therapy. Node-negative patients do not benefit from axillary surgery, and they may suffer from complications regardless of the type of surgery performed, i.e., SNB or ALND [9]. However, the incidence of SN metastasis has been reported to be 33.2% in invasive breast cancer [21].

SNB has been used as standard method for the assessment of axillary status since the early 2000s, and usually, a SNB will be followed by an axillary dissection in case of SN metastases, but for the last 5 years and in recent publications, ALND has been questioned in patients with metastatic SN due to the encouraging survival results for patient not undergoing axillary surgery [22]. This has led to calls for more conservative management of the axilla in early breast cancer, and there is still continued debate about the role of axillary dissection in this patient population [23].

In this study, we observed that tumor size is an independent predictive factor for positive SN status, where SN metastases were observed in 367 patients with T1 (24.3%). Capdet et al. showed in a study involving 1416 patients that SN metastases were detected in 368 patients (26%) with T1 cancer, and young age, tumor size and location, histological type, histological grade, and lymph vascular invasion appeared to be significant risk factors of SN involvement [24]. Viale et al. showed in a study involving more than 4000 patients that tumor size and peritumoral vascular invasion emerged as the most powerful independent predictors for SN metastases [21].

In our study, the risk of SN metastases was not influenced by histological grade (Table 2); other studies have shown that the risk of SN metastases increased not only depending on tumor size but also on the histological grade and the patient age. Mustafa et al. showed in a study involving more than 2000 patients with T1 tumors that histological grades II–III in women before the age of 40 years had higher incidence of sentinel node involvement compared with histological grade I [25].

We observed in this study that the risk of SN involvement was low in tumors of rare type, e.g., medullary breast cancer. However, all rare tumors were merged in one sub-group in this study as these types were rare and separate analyses were difficult due to poor statistical power (Table 2).

We observed in our study that the strongest independent predictor of SN involvement was lymphovascular invasion (3.74; 2.66–5.27) followed by, in order of significance, size of the tumor (2.56; 1.07–6.09) and multifocality (1.90; 1.45–2.47), while Gajdos et al. showed in a study which involved 850 consecutive patients who underwent ALND for T1 breast cancer that axillary lymph node metastases were most significantly related to lymphatic invasion in the primary tumor, followed by tumor size and patient age [26]. Yoshihara et al. has showed in their evaluation of 1300 patients that lymphovascular invasion and tumor size emerged as the most powerful independent predictors of ALN metastases, followed by the location of the tumor in the breast and the presence of multiple foci [27]. However, the usefulness of lymphovascular involvement in decision making before surgery is of limited clinical value as this factor is not known until the final pathological report is available.

Mammography screening for breast cancer becomes more prevalent; improvements in imaging and new techniques make breast tumors easier to be found at smaller sizes than before [28]. In this study, we observed that breast cancer which is detected by mammography screening had lower risk for metastatic involvement of the sentinel nodes. This is probably due to many different factors but most possibly because of early detection of invasive tumors with small size less than 10 mm which is in turn associated with lower risk for SN metastases.

The possibility of metastatic involvement of SN in breast cancer with negative hormonal status particularly estrogen receptor status has not been established clearly compared with receptor-positive tumor. Our findings indicate that the risk of SN metastases is low in tumors with negative hormonal status for estrogen (0.64; 0.42–0.99). Mattes et al. observed in their study including 7274 patients with T1–T3 infiltrating ductal cancer that HR−/HER2− cancers had a significantly lower risk (OR 0.686) of nodal positivity than the HR+/HER2− subtype [29]. Similarly has Ugras et al. showed in their study involving 11,596 patients that nodal metastases were least frequent in triple negative (TN) cancers compared with other subtypes [30].

The results of this study showed that it is possible to identify patients with invasive breast cancer with a high risk of metastatic involvement of the sentinel nodes. This knowledge is useful in clinical practice and it might help in order to improve planning for surgical or systemic therapy. Furthermore, this study might help in identifying patients with a high probability of node-negative tumor where the SN procedure may possibly be omitted, although it is still very difficult to identify and select cases defiantly as the most powerful predictors for metastases to SN according to many studies are those which are available after histopathological examination such as lymphovascular invasion.

## Conclusions

We conclude that SN metastasis is less likely to occur in women with invasive breast cancer diagnosed by screening mammogram and in tumors with negative estrogen status. Tumors larger than 20 mm, multifocality, or lymphovascular invasion are also factors associated with higher risk for SN metastases.

## Abbreviations

ALND: Axillary lymph node dissection
CI: Confidence interval
FISH: Fluorescent in situ hybridization
HER2: Human epidermal growth factor receptor 2
HR: Hormone receptor
IHC: Immunohistochemistry
INCA: Information Network for Cancer Care
ITC: Isolated tumor cell
LU-Dnr: Lund document number
NHG: Nottingham histological grading
OR: Odds ratio
RCC-Syd: Regional Cancer Center in Southern Sweden
SN: Sentinel node
SNB: Sentinel node biopsy
SPSS: Statistical Package for the Social Sciences
TN: Triple negative
TNM: Tumor lymph node metastasis
WHO: World Health Organization
